# Improved limit on the branching fraction of the rare decay ${{K} ^0_{\mathrm { \scriptscriptstyle S}}} \rightarrow \mu ^+\mu ^-$

**DOI:** 10.1140/epjc/s10052-017-5230-x

**Published:** 2017-10-13

**Authors:** R. Aaij, B. Adeva, M. Adinolfi, Z. Ajaltouni, S. Akar, J. Albrecht, F. Alessio, M. Alexander, S. Ali, G. Alkhazov, P. Alvarez Cartelle, A. A. Alves, S. Amato, S. Amerio, Y. Amhis, L. An, L. Anderlini, G. Andreassi, M. Andreotti, J. E. Andrews, R. B. Appleby, F. Archilli, P. d’Argent, J. Arnau Romeu, A. Artamonov, M. Artuso, E. Aslanides, G. Auriemma, M. Baalouch, I. Babuschkin, S. Bachmann, J. J. Back, A. Badalov, C. Baesso, S. Baker, V. Balagura, W. Baldini, A. Baranov, R. J. Barlow, C. Barschel, S. Barsuk, W. Barter, F. Baryshnikov, M. Baszczyk, V. Batozskaya, V. Battista, A. Bay, L. Beaucourt, J. Beddow, F. Bedeschi, I. Bediaga, A. Beiter, L. J. Bel, V. Bellee, N. Belloli, K. Belous, I. Belyaev, E. Ben-Haim, G. Bencivenni, S. Benson, S. Beranek, A. Berezhnoy, R. Bernet, A. Bertolin, C. Betancourt, F. Betti, M.-O. Bettler, M. van Beuzekom, Ia. Bezshyiko, S. Bifani, P. Billoir, A. Birnkraut, A. Bitadze, A. Bizzeti, T. Blake, F. Blanc, J. Blouw, S. Blusk, V. Bocci, T. Boettcher, A. Bondar, N. Bondar, W. Bonivento, I. Bordyuzhin, A. Borgheresi, S. Borghi, M. Borisyak, M. Borsato, F. Bossu, M. Boubdir, T. J. V. Bowcock, E. Bowen, C. Bozzi, S. Braun, T. Britton, J. Brodzicka, E. Buchanan, C. Burr, A. Bursche, J. Buytaert, S. Cadeddu, R. Calabrese, M. Calvi, M. Calvo Gomez, A. Camboni, P. Campana, D. H. Campora Perez, L. Capriotti, A. Carbone, G. Carboni, R. Cardinale, A. Cardini, P. Carniti, L. Carson, K. Carvalho Akiba, G. Casse, L. Cassina, L. Castillo Garcia, M. Cattaneo, G. Cavallero, R. Cenci, D. Chamont, M. Charles, Ph. Charpentier, G. Chatzikonstantinidis, M. Chefdeville, S. Chen, S. F. Cheung, V. Chobanova, M. Chrzaszcz, A. Chubykin, X. Cid Vidal, G. Ciezarek, P. E. L. Clarke, M. Clemencic, H. V. Cliff, J. Closier, V. Coco, J. Cogan, E. Cogneras, V. Cogoni, L. Cojocariu, P. Collins, A. Comerma-Montells, A. Contu, A. Cook, G. Coombs, S. Coquereau, G. Corti, M. Corvo, C. M. Costa Sobral, B. Couturier, G. A. Cowan, D. C. Craik, A. Crocombe, M. Cruz Torres, R. Currie, C. D’Ambrosio, F. Da Cunha Marinho, E. Dall’Occo, J. Dalseno, A. Davis, K. De Bruyn, S. De Capua, M. De Cian, J. M. De Miranda, L. De Paula, M. De Serio, P. De Simone, C. T. Dean, D. Decamp, M. Deckenhoff, L. Del Buono, H.-P. Dembinski, M. Demmer, A. Dendek, D. Derkach, O. Deschamps, F. Dettori, B. Dey, A. Di Canto, P. Di Nezza, H. Dijkstra, F. Dordei, M. Dorigo, A. Dosil Suárez, A. Dovbnya, K. Dreimanis, L. Dufour, G. Dujany, K. Dungs, P. Durante, R. Dzhelyadin, M. Dziewiecki, A. Dziurda, A. Dzyuba, N. Déléage, S. Easo, M. Ebert, U. Egede, V. Egorychev, S. Eidelman, S. Eisenhardt, U. Eitschberger, R. Ekelhof, L. Eklund, S. Ely, S. Esen, H. M. Evans, T. Evans, A. Falabella, N. Farley, S. Farry, R. Fay, D. Fazzini, D. Ferguson, G. Fernandez, A. Fernandez Prieto, F. Ferrari, F. Ferreira Rodrigues, M. Ferro-Luzzi, S. Filippov, R. A. Fini, M. Fiore, M. Fiorini, M. Firlej, C. Fitzpatrick, T. Fiutowski, F. Fleuret, K. Fohl, M. Fontana, F. Fontanelli, D. C. Forshaw, R. Forty, V. Franco Lima, M. Frank, C. Frei, J. Fu, W. Funk, E. Furfaro, C. Färber, A. Gallas Torreira, D. Galli, S. Gallorini, S. Gambetta, M. Gandelman, P. Gandini, Y. Gao, L. M. Garcia Martin, J. García Pardiñas, J. Garra Tico, L. Garrido, P. J. Garsed, D. Gascon, C. Gaspar, L. Gavardi, G. Gazzoni, D. Gerick, E. Gersabeck, M. Gersabeck, T. Gershon, Ph. Ghez, S. Gianì, V. Gibson, O. G. Girard, L. Giubega, K. Gizdov, V. V. Gligorov, D. Golubkov, A. Golutvin, A. Gomes, I. V. Gorelov, C. Gotti, E. Govorkova, R. Graciani Diaz, L. A. Granado Cardoso, E. Graugés, E. Graverini, G. Graziani, A. Grecu, R. Greim, P. Griffith, L. Grillo, B. R. Gruberg Cazon, O. Grünberg, E. Gushchin, Yu. Guz, T. Gys, C. Göbel, T. Hadavizadeh, C. Hadjivasiliou, G. Haefeli, C. Haen, S. C. Haines, B. Hamilton, X. Han, S. Hansmann-Menzemer, N. Harnew, S. T. Harnew, J. Harrison, M. Hatch, J. He, T. Head, A. Heister, K. Hennessy, P. Henrard, L. Henry, E. van Herwijnen, M. Heß, A. Hicheur, D. Hill, C. Hombach, P. H. Hopchev, Z.-C. Huard, W. Hulsbergen, T. Humair, M. Hushchyn, D. Hutchcroft, M. Idzik, P. Ilten, R. Jacobsson, J. Jalocha, E. Jans, A. Jawahery, F. Jiang, M. John, D. Johnson, C. R. Jones, C. Joram, B. Jost, N. Jurik, S. Kandybei, M. Karacson, J. M. Kariuki, S. Karodia, M. Kecke, M. Kelsey, M. Kenzie, T. Ketel, E. Khairullin, B. Khanji, C. Khurewathanakul, T. Kirn, S. Klaver, K. Klimaszewski, T. Klimkovich, S. Koliiev, M. Kolpin, I. Komarov, R. Kopecna, P. Koppenburg, A. Kosmyntseva, S. Kotriakhova, M. Kozeiha, L. Kravchuk, M. Kreps, P. Krokovny, F. Kruse, W. Krzemien, W. Kucewicz, M. Kucharczyk, V. Kudryavtsev, A. K. Kuonen, K. Kurek, T. Kvaratskheliya, D. Lacarrere, G. Lafferty, A. Lai, G. Lanfranchi, C. Langenbruch, T. Latham, C. Lazzeroni, R. Le Gac, J. van Leerdam, A. Leflat, J. Lefrançois, R. Lefèvre, F. Lemaitre, E. Lemos Cid, O. Leroy, T. Lesiak, B. Leverington, T. Li, Y. Li, Z. Li, T. Likhomanenko, R. Lindner, F. Lionetto, X. Liu, D. Loh, I. Longstaff, J. H. Lopes, D. Lucchesi, M. Lucio Martinez, H. Luo, A. Lupato, E. Luppi, O. Lupton, A. Lusiani, X. Lyu, F. Machefert, F. Maciuc, O. Maev, K. Maguire, S. Malde, A. Malinin, T. Maltsev, G. Manca, G. Mancinelli, P. Manning, J. Maratas, J. F. Marchand, U. Marconi, C. Marin Benito, M. Marinangeli, P. Marino, J. Marks, G. Martellotti, M. Martin, M. Martinelli, D. Martinez Santos, F. Martinez Vidal, D. Martins Tostes, L. M. Massacrier, A. Massafferri, R. Matev, A. Mathad, Z. Mathe, C. Matteuzzi, A. Mauri, E. Maurice, B. Maurin, A. Mazurov, M. McCann, A. McNab, R. McNulty, B. Meadows, F. Meier, D. Melnychuk, M. Merk, A. Merli, E. Michielin, D. A. Milanes, M.-N. Minard, D. S. Mitzel, A. Mogini, J. Molina Rodriguez, I. A. Monroy, S. Monteil, M. Morandin, M. J. Morello, O. Morgunova, J. Moron, A. B. Morris, R. Mountain, F. Muheim, M. Mulder, M. Mussini, D. Müller, J. Müller, K. Müller, V. Müller, P. Naik, T. Nakada, R. Nandakumar, A. Nandi, I. Nasteva, M. Needham, N. Neri, S. Neubert, N. Neufeld, M. Neuner, T. D. Nguyen, C. Nguyen-Mau, S. Nieswand, R. Niet, N. Nikitin, T. Nikodem, A. Nogay, A. Novoselov, D. P. O’Hanlon, A. Oblakowska-Mucha, V. Obraztsov, S. Ogilvy, R. Oldeman, C. J. G. Onderwater, A. Ossowska, J. M. Otalora Goicochea, P. Owen, A. Oyanguren, P. R. Pais, A. Palano, M. Palutan, A. Papanestis, M. Pappagallo, L. L. Pappalardo, C. Pappenheimer, W. Parker, C. Parkes, G. Passaleva, A. Pastore, M. Patel, C. Patrignani, A. Pearce, A. Pellegrino, G. Penso, M. Pepe Altarelli, S. Perazzini, P. Perret, L. Pescatore, K. Petridis, A. Petrolini, A. Petrov, M. Petruzzo, E. Picatoste Olloqui, B. Pietrzyk, M. Pikies, D. Pinci, A. Pistone, A. Piucci, V. Placinta, S. Playfer, M. Plo Casasus, T. Poikela, F. Polci, M. Poli Lener, A. Poluektov, I. Polyakov, E. Polycarpo, G. J. Pomery, S. Ponce, A. Popov, D. Popov, B. Popovici, S. Poslavskii, C. Potterat, E. Price, J. Prisciandaro, C. Prouve, V. Pugatch, A. Puig Navarro, G. Punzi, W. Qian, R. Quagliani, B. Rachwal, J. H. Rademacker, M. Rama, M. Ramos Pernas, M. S. Rangel, I. Raniuk, F. Ratnikov, G. Raven, F. Redi, S. Reichert, A. C. dos Reis, C. Remon Alepuz, V. Renaudin, S. Ricciardi, S. Richards, M. Rihl, K. Rinnert, V. Rives Molina, P. Robbe, A. B. Rodrigues, E. Rodrigues, J. A. Rodriguez Lopez, P. Rodriguez Perez, A. Rogozhnikov, S. Roiser, A. Rollings, V. Romanovskiy, A. Romero Vidal, J. W. Ronayne, M. Rotondo, M. S. Rudolph, T. Ruf, P. Ruiz Valls, J. J. Saborido Silva, E. Sadykhov, N. Sagidova, B. Saitta, V. Salustino Guimaraes, D. Sanchez Gonzalo, C. Sanchez Mayordomo, B. Sanmartin Sedes, R. Santacesaria, C. Santamarina Rios, M. Santimaria, E. Santovetti, A. Sarti, C. Satriano, A. Satta, D. M. Saunders, D. Savrina, S. Schael, M. Schellenberg, M. Schiller, H. Schindler, M. Schlupp, M. Schmelling, T. Schmelzer, B. Schmidt, O. Schneider, A. Schopper, H. F. Schreiner, K. Schubert, M. Schubiger, M.-H. Schune, R. Schwemmer, B. Sciascia, A. Sciubba, A. Semennikov, A. Sergi, N. Serra, J. Serrano, L. Sestini, P. Seyfert, M. Shapkin, I. Shapoval, Y. Shcheglov, T. Shears, L. Shekhtman, V. Shevchenko, B. G. Siddi, R. Silva Coutinho, L. Silva de Oliveira, G. Simi, S. Simone, M. Sirendi, N. Skidmore, T. Skwarnicki, E. Smith, I. T. Smith, J. Smith, M. Smith, l. Soares Lavra, M. D. Sokoloff, F. J. P. Soler, B. Souza De Paula, B. Spaan, P. Spradlin, S. Sridharan, F. Stagni, M. Stahl, S. Stahl, P. Stefko, S. Stefkova, O. Steinkamp, S. Stemmle, O. Stenyakin, H. Stevens, S. Stoica, S. Stone, B. Storaci, S. Stracka, M. E. Stramaglia, M. Straticiuc, U. Straumann, L. Sun, W. Sutcliffe, K. Swientek, V. Syropoulos, M. Szczekowski, T. Szumlak, S. T’Jampens, A. Tayduganov, T. Tekampe, G. Tellarini, F. Teubert, E. Thomas, J. van Tilburg, M. J. Tilley, V. Tisserand, M. Tobin, S. Tolk, L. Tomassetti, D. Tonelli, S. Topp-Joergensen, F. Toriello, R. Tourinho Jadallah Aoude, E. Tournefier, S. Tourneur, K. Trabelsi, M. Traill, M. T. Tran, M. Tresch, A. Trisovic, A. Tsaregorodtsev, P. Tsopelas, A. Tully, N. Tuning, A. Ukleja, A. Ustyuzhanin, U. Uwer, C. Vacca, V. Vagnoni, A. Valassi, S. Valat, G. Valenti, R. Vazquez Gomez, P. Vazquez Regueiro, S. Vecchi, M. van Veghel, J. J. Velthuis, M. Veltri, G. Veneziano, A. Venkateswaran, T. A. Verlage, M. Vernet, M. Vesterinen, J. V. Viana Barbosa, B. Viaud, D. Vieira, M. Vieites Diaz, H. Viemann, X. Vilasis-Cardona, M. Vitti, V. Volkov, A. Vollhardt, B. Voneki, A. Vorobyev, V. Vorobyev, C. Voß, J. A. de Vries, C. Vázquez Sierra, R. Waldi, C. Wallace, R. Wallace, J. Walsh, J. Wang, D. R. Ward, H. M. Wark, N. K. Watson, D. Websdale, A. Weiden, M. Whitehead, J. Wicht, G. Wilkinson, M. Wilkinson, M. Williams, M. P. Williams, M. Williams, T. Williams, F. F. Wilson, J. Wimberley, M. A. Winn, J. Wishahi, W. Wislicki, M. Witek, G. Wormser, S. A. Wotton, K. Wraight, K. Wyllie, Y. Xie, Z. Xu, Z. Yang, Z. Yang, Y. Yao, H. Yin, J. Yu, X. Yuan, O. Yushchenko, K. A. Zarebski, M. Zavertyaev, L. Zhang, Y. Zhang, A. Zhelezov, Y. Zheng, X. Zhu, V. Zhukov, S. Zucchelli, LHCb Collaboration

**Affiliations:** 1 Centro Brasileiro de Pesquisas Físicas (CBPF), Rio de Janeiro, Brazil; 2 Universidade Federal do Rio de Janeiro (UFRJ), Rio de Janeiro, Brazil; 3 Center for High Energy Physics, Tsinghua University, Beijing, China; 4 LAPP, Université Savoie Mont-Blanc, CNRS/IN2P3, Annecy-Le-Vieux, France; 5 Clermont Université, Université Blaise Pascal, CNRS/IN2P3, LPC, Clermont-Ferrand, France; 6 CPPM, Aix-Marseille Université, CNRS/IN2P3, Marseille, France; 7 LAL, Université Paris-Sud, CNRS/IN2P3, Orsay, France; 8 LPNHE, Université Pierre et Marie Curie, Université Paris Diderot, CNRS/IN2P3, Paris, France; 9 I. Physikalisches Institut, RWTH Aachen University, Aachen, Germany; 10 Fakultät Physik, Technische Universität Dortmund, Dortmund, Germany; 11 Max-Planck-Institut für Kernphysik (MPIK), Heidelberg, Germany; 12 Physikalisches Institut, Ruprecht-Karls-Universität Heidelberg, Heidelberg, Germany; 13 School of Physics, University College Dublin, Dublin, Ireland; 14 Sezione INFN di Bari, Bari, Italy; 15 Sezione INFN di Bologna, Bologna, Italy; 16 Sezione INFN di Cagliari, Cagliari, Italy; 17 Universita e INFN Ferrara, Ferrara, Italy; 18 Sezione INFN di Firenze, Florence, Italy; 19 Laboratori Nazionali dell’INFN di Frascati, Frascati, Italy; 20 Sezione INFN di Genova, Genoa, Italy; 21 Universita and INFN Milano-Bicocca, Milan, Italy; 22 Sezione di Milano, Milan, Italy; 23 Sezione INFN di Padova, Padua, Italy; 24 Sezione INFN di Pisa, Pisa, Italy; 25 Sezione INFN di Roma Tor Vergata, Rome, Italy; 26 Sezione INFN di Roma La Sapienza, Rome, Italy; 27 Henryk Niewodniczanski Institute of Nuclear Physics Polish Academy of Sciences, Kraków, Poland; 28 Faculty of Physics and Applied Computer Science, AGH-University of Science and Technology, Kraków, Poland; 29 National Center for Nuclear Research (NCBJ), Warsaw, Poland; 30 Horia Hulubei National Institute of Physics and Nuclear Engineering, Bucharest-Magurele, Romania; 31 Petersburg Nuclear Physics Institute (PNPI), Gatchina, Russia; 32 Institute of Theoretical and Experimental Physics (ITEP), Moscow, Russia; 33 Institute of Nuclear Physics, Moscow State University (SINP MSU), Moscow, Russia; 34 Institute for Nuclear Research of the Russian Academy of Sciences (INR RAN), Moscow, Russia; 35 Yandex School of Data Analysis, Moscow, Russia; 36 Budker Institute of Nuclear Physics (SB RAS), Novosibirsk, Russia; 37 Institute for High Energy Physics (IHEP), Protvino, Russia; 38 ICCUB, Universitat de Barcelona, Barcelona, Spain; 39 Universidad de Santiago de Compostela, Santiago de Compostela, Spain; 40 European Organization for Nuclear Research (CERN), Geneva, Switzerland; 41 Institute of Physics, Ecole Polytechnique Fédérale de Lausanne (EPFL), Lausanne, Switzerland; 42 Physik-Institut, Universität Zürich, Zurich, Switzerland; 43 Nikhef National Institute for Subatomic Physics, Amsterdam, The Netherlands; 44 Nikhef National Institute for Subatomic Physics and VU University Amsterdam, Amsterdam, The Netherlands; 45 NSC Kharkiv Institute of Physics and Technology (NSC KIPT), Kharkiv, Ukraine; 46 Institute for Nuclear Research of the National Academy of Sciences (KINR), Kyiv, Ukraine; 47 University of Birmingham, Birmingham, UK; 48 H.H. Wills Physics Laboratory, University of Bristol, Bristol, UK; 49 Cavendish Laboratory, University of Cambridge, Cambridge, UK; 50 Department of Physics, University of Warwick, Coventry, UK; 51 STFC Rutherford Appleton Laboratory, Didcot, UK; 52 School of Physics and Astronomy, University of Edinburgh, Edinburgh, UK; 53 School of Physics and Astronomy, University of Glasgow, Glasgow, UK; 54 Oliver Lodge Laboratory, University of Liverpool, Liverpool, UK; 55 Imperial College London, London, UK; 56 School of Physics and Astronomy, University of Manchester, Manchester, UK; 57 Department of Physics, University of Oxford, Oxford, UK; 58 Massachusetts Institute of Technology, Cambridge, MA USA; 59 University of Cincinnati, Cincinnati, OH USA; 60 University of Maryland, College Park, MD USA; 61 Syracuse University, Syracuse, NY USA; 62 Pontifícia Universidade Católica do Rio de Janeiro (PUC-Rio), Rio de Janeiro, Brazil; 63 University of Chinese Academy of Sciences, Beijing, China; 64 School of Physics and Technology, Wuhan University, Wuhan, China; 65 Institute of Particle Physics, Central China Normal University, Wuhan, Hubei China; 66 Departamento de Fisica, Universidad Nacional de Colombia, Bogotá, Colombia; 67 Institut für Physik, Universität Rostock, Rostock, Germany; 68 National Research Centre Kurchatov Institute, Moscow, Russia; 69 Instituto de Fisica Corpuscular, Centro Mixto Universidad de Valencia-CSIC, Valencia, Spain; 70 Van Swinderen Institute, University of Groningen, Groningen, The Netherlands; 71 CERN, 1211 Geneva 23, Switzerland

## Abstract

A search for the decay ${{K} ^0_{\mathrm { \scriptscriptstyle S}}} \rightarrow \mu ^+\mu ^-$ is performed, based on a data sample of proton-proton collisions corresponding to an integrated luminosity of $3\,\text{ fb }^{-1}$, collected by the LHCb experiment at centre-of-mass energies of 7 and 8$\mathrm {\,TeV}$. The observed yield is consistent with the background-only hypothesis, yielding a limit on the branching fraction of $\mathcal{B}({{K} ^0_{\mathrm { \scriptscriptstyle S}}} \rightarrow \mu ^+\mu ^-) < 0.8~(1.0) \times 10^{-9}$ at $90\%~(95\%)$ confidence level. This result improves the previous upper limit on the branching fraction by an order of magnitude.

## Introduction

In the Standard Model (SM), the unobserved ${{K} ^0_{\mathrm { \scriptscriptstyle S}}} \rightarrow \mu ^+\mu ^-$ decay proceeds only through a Flavour-Changing Neutral Current (FCNC) transition, which cannot occur at tree level. It is further suppressed by the small amount of $C\!P$ violation in kaon decays, since the S-wave component of the decay is forbidden when $C\!P$ is conserved. In the SM, the decay amplitude is expected to be dominated by long distance contributions, which can be constrained using the observed decays ${{K} ^0_{\mathrm { \scriptscriptstyle S}}} \!\rightarrow \gamma \gamma$ and ${{K} ^0_{\mathrm { \scriptscriptstyle L}}} \!\rightarrow {{\pi } ^0} \gamma \gamma$, leading to the prediction for the branching fraction $\mathcal{B}({{K} ^0_{\mathrm { \scriptscriptstyle S}}} \rightarrow \mu ^+\mu ^-) = (5.0 \pm 1.5) \times 10^{-12}$ [1, 2]. The predicted branching fraction for the ${K} ^0_{\mathrm { \scriptscriptstyle L}}$ decay is $(6.85 \pm 0.32) \times 10^{-9}$ [3], in excellent agreement with the experimental world average ${\mathcal {B}} ({{K} ^0_{\mathrm { \scriptscriptstyle L}}} \!\rightarrow {\mu ^+\mu ^-} ) = (6.84 \pm 0.11) \times 10^{-9}$ [4]. The prediction for ${{K} ^0_{\mathrm { \scriptscriptstyle S}}} \rightarrow \mu ^+\mu ^-$ is currently being updated with a dispersive treatment, which leads to sizeable corrections in other ${K} ^0_{\mathrm { \scriptscriptstyle S}}$ leptonic decays [5].

Due to its suppression in the SM, the ${{K} ^0_{\mathrm { \scriptscriptstyle S}}} \rightarrow \mu ^+\mu ^-$ decay is sensitive to possible contributions from dynamics beyond the SM, notably from light scalars with $C\!P$-violating Yukawa couplings [1]. Contributions up to one order of magnitude above the SM branching fraction expectation naturally arise in many models and are compatible with the present bounds from other FCNC processes. An upper limit on $\mathcal{B}({{K} ^0_{\mathrm { \scriptscriptstyle S}}} \rightarrow \mu ^+\mu ^-)$ close to $10^{-11}$ could be translated into model-independent bounds on the $C\!P$-violating phase of the $s\!\rightarrow d{\ell ^+} {\ell ^-}$ amplitude [2]. This would be very useful to discriminate between scenarios beyond the SM if other modes, such as ${{K} ^+} \!\rightarrow {{\pi } ^+} {\nu } {\overline{\nu }}$, indicate a non-SM enhancement.

The current experimental limit, ${\mathcal {B}} ({{K} ^0_{\mathrm { \scriptscriptstyle S}}} \rightarrow \mu ^+\mu ^-) < 9 \times 10^{-9}$ at $90\%$ confidence level (CL), was obtained using *pp* collision data corresponding to $1.0\,\text{ fb }^{-1}$ of integrated luminosity at a centre-of-mass energy $\sqrt{s}=7~{\mathrm {\,TeV}}$, collected with the LHCb detector in 2011 [6]. This result improved the previous upper limit [7] but is still three orders of magnitude above the predicted SM level.

In this paper, an update of the search for the ${{K} ^0_{\mathrm { \scriptscriptstyle S}}} \rightarrow \mu ^+\mu ^-$ decay is reported. Its branching fraction is measured using the known ${{K} ^0_{\mathrm { \scriptscriptstyle S}}} \rightarrow \pi ^+\pi ^-$ decay as normalisation. The analysis is performed on a data sample corresponding to $2\,\text{ fb }^{-1}$ of integrated luminosity at $\sqrt{s}=8~{\mathrm {\,TeV}}$, collected in 2012, and the result is combined with that from the previous LHCb analysis [6]. Besides the gain in statistical precision due to the larger data sample, the sensitivity is noticeably increased with respect to the previous result due to a higher trigger efficiency, as well as other improvements to the analysis that are discussed in the following sections.

An overview on how ${{K} ^0_{\mathrm { \scriptscriptstyle S}}} \rightarrow \mu ^+\mu ^-$ decays are detected and triggered in LHCb is given in Sect. 2, while the strategy for this measurement is outlined in Sect. 3. Details of background suppression and the resulting sensitivity are given in Sects. 4 and 5, respectively. The final result, taking into account the systematic uncertainties discussed in Sect. 6, is given in Sect. 7.

## ${K} ^0_{\mathrm { \scriptscriptstyle S}}$ decays in LHCb

The LHCb detector [8, 9] is a single-arm forward spectrometer covering the pseudorapidity range $$2<\eta <5$$, designed for the study of particles containing $$b $$ or $$c $$ quarks. The detector includes a high-precision tracking system consisting of a silicon-strip vertex locator (VELO) surrounding the *pp* interaction region, a large-area silicon-strip detector located upstream of a dipole magnet with a bending power of about $$4{\mathrm {\,Tm}}$$, and three stations of silicon-strip detectors and straw drift tubes placed downstream of the magnet. The tracking system provides a measurement of momentum, $$p$$, of charged particles with a relative uncertainty that varies from $$0.5\%$$ at low momentum to $$1.0\%$$ at $$200\,{\mathrm {\,GeV\!/}c} $$. The minimum distance of a track to a primary vertex (PV), the impact parameter (IP), is measured with a resolution of $$(15+29/p_{\mathrm { T}})\,{\,\upmu \mathrm {m}} $$, where $$p_{\mathrm { T}}$$ is the component of the momentum transverse to the beam, in $${\mathrm {\,GeV\!/}c}$$. Different types of charged hadrons are distinguished using information from two ring-imaging Cherenkov detectors  (RICH). Photons, electrons and hadrons are identified by a calorimeter system consisting of scintillating-pad and preshower detectors, an electromagnetic calorimeter and a hadronic calorimeter. Muons are identified by five stations which alternate layers of iron and multiwire proportional chambers.

The online event selection is performed by the trigger [10], which consists of a hardware stage, based on information from the calorimeter and muon systems, followed by a two-step software stage, which applies a full event reconstruction. Candidates are subsequently classified as TOS, if the event is triggered on the signal candidate, or TIS, if triggered by other activities in the detector, independently of signal. Only candidates that are classified as TOS at each trigger stage are used to search for ${{K} ^0_{\mathrm { \scriptscriptstyle S}}} \rightarrow \mu ^+\mu ^-$ decays.

The trigger selection constitutes the main limitation to the efficiency for detecting ${K} ^0_{\mathrm { \scriptscriptstyle S}}$ decays. A muon is only selected at the hardware stage when it is detected in all muon stations and a rough momentum estimation is provided. Trigger requirements at this stage imply a momentum larger than about $5\,{\mathrm {\,GeV\!/}c}$, and a $p_{\mathrm { T}}$ above $1.76\,{\mathrm {\,GeV\!/}c}$. These thresholds have an efficiency of order $1\%$ for ${{K} ^0_{\mathrm { \scriptscriptstyle S}}} \rightarrow \mu ^+\mu ^-$ decays.

In the first step of the software trigger, all charged particles with $p_{\mathrm { T}} >500\,{\mathrm {\,MeV\!/}c}$ are reconstructed. At this stage most signal decays are triggered either by requiring a reconstructed track loosely identified as a muon [10, 11], with $\text {IP}>0.1\,\mathrm { \,mm}$ and $p_{\mathrm { T}} >1.0\,{\mathrm {\,GeV\!/}c}$, or by finding two oppositely charged muon candidates forming a detached secondary vertex (SV). Since these two categories, hereafter referred to as TOS$_{\mu }$ and TOS$_{\mu \mu }$, induce different kinematic biases on the signal and background candidates, the analysis steps described below are performed independently on each category. The two categories are made mutually exclusive by applying the TOS$_{\mu \mu }$ selection only to candidates not already selected by TOS$_{\mu }$.

In the second software trigger stage, an offline-quality event reconstruction is performed. Signal candidates are selected requiring a dimuon with $p_{\mathrm { T}} >600\,{\mathrm {\,MeV\!/}c}$ detached from the primary vertex, with both tracks having $p_{\mathrm { T}} >300\,{\mathrm {\,MeV\!/}c}$. In the 2011 data taking, the dimuon mass was required to be larger than $1\,{\mathrm {\,GeV\!/}c^2}$ in the second software trigger stage. This excluded the ${K} ^0_{\mathrm { \scriptscriptstyle S}}$ region, making the use of TIS candidates necessary. Due to the trigger reoptimisation, no mass requirements were applied during 2012 and a lower $p_{\mathrm { T}}$ threshold for reconstructed tracks was used. According to simulation, these changes improve the trigger efficiency over the previous analysis [6] by about a factor 2.5.

Due to its large and well-known branching fraction and its similar topology, the ${{K} ^0_{\mathrm { \scriptscriptstyle S}}} \rightarrow \pi ^+\pi ^-$ decay is taken as the normalisation mode. A large sample of candidates is obtained from an unbiased trigger, which does not apply any selection requirement.

Despite the low trigger efficiency, the study detailed in this paper profits from the unprecedented number of ${K} ^0_{\mathrm { \scriptscriptstyle S}}$ produced at the LHC, $\mathcal {O}(10^{13})$ per $\text{ fb }^{-1}$ of integrated luminosity within the LHCb acceptance, and from the fact that about $40\%$ of these ${K} ^0_{\mathrm { \scriptscriptstyle S}}$ decays occur inside the VELO region. For such decays, the ${K} ^0_{\mathrm { \scriptscriptstyle S}}$ invariant mass is reconstructed with a resolution of about $4\,{\mathrm {\,MeV\!/}c^2}$.

The analysis makes use of large samples of simulated collisions containing a signal decay, or background decays which can be reconstructed as the signal, and contaminate the $\mu \mu$ invariant mass distribution, such as ${{K} ^0_{\mathrm { \scriptscriptstyle S}}} \rightarrow \pi ^+\pi ^-$ or ${{K} ^0_{\mathrm { \scriptscriptstyle S}}} \rightarrow \pi ^+\mu ^-\bar{\nu }_\mu$.^1^ In the simulation, *pp* collisions are generated using Pythia  [12, 13] with a specific LHCb configuration [14]. Decays of hadronic particles are described by EvtGen  [15], in which final-state radiation is generated using Photos  [16]. The interaction of the generated particles with the detector, and its response, are implemented using the Geant4 toolkit [17, 18] as described in Ref. [19].

## Selection and search strategy

Common offline preselection criteria are applied to ${{K} ^0_{\mathrm { \scriptscriptstyle S}}} \rightarrow \mu ^+\mu ^-$ and ${{K} ^0_{\mathrm { \scriptscriptstyle S}}} \rightarrow \pi ^+\pi ^-$ candidates to cancel many systematic effects in the ratio. Candidates are required to decay in the VELO region, where the best ${K} ^0_{\mathrm { \scriptscriptstyle S}}$ mass resolution is achieved. The two reconstructed tracks must have momentum smaller than $100\,{\mathrm {\,GeV\!/}c}$ and quality requirements are set on the track and secondary vertex fits. The SV must be well detached from the PV by requiring the ${K} ^0_{\mathrm { \scriptscriptstyle S}}$ decay time to be larger than $8.95\,$ps, $10\%$ of the ${K} ^0_{\mathrm { \scriptscriptstyle S}}$ mean lifetime. The ${K} ^0_{\mathrm { \scriptscriptstyle S}}$ IP must be less than $0.4\,\mathrm { \,mm}$, while the two charged tracks are required to be incompatible with originating from any PV, with IP $\chi ^2$, defined as the difference of the $\chi ^2$ of the PV fit obtained with and without the considered track, to be larger than 100.

Decays of  baryons to ${p} {{\pi } ^-}$ are suppressed by removing candidates close to the expected ellipses in the Armenteros–Podolanski (AP) plane [20]. In this plane the $p_{\mathrm { T}}$ of the final-state particles under the pion mass hypothesis is plotted versus the longitudinal momentum asymmetry, defined as $\alpha = (p_L ^+ - p_L ^-)/(p_L ^+ + p_L ^-)$, where $p_L ^\pm$ is the longitudinal momentum of the charged tracks. Both $p_{\mathrm { T}}$ and $p_L$ are considered with respect to the direction of the mother particle. The ${K} ^0_{\mathrm { \scriptscriptstyle S}}$ decays are symmetrically distributed on the AP plane while  decays produce two ellipses at low $p_{\mathrm { T}}$ and $|\alpha |\sim 0.7$. A kaon veto, based on the response of the RICH detector, is used to suppress ${{K} ^{*0}} \!\rightarrow {{K} ^+} {{\pi } ^-}$ decays and other possible final states including a charged kaon.

**Fig. 1:**
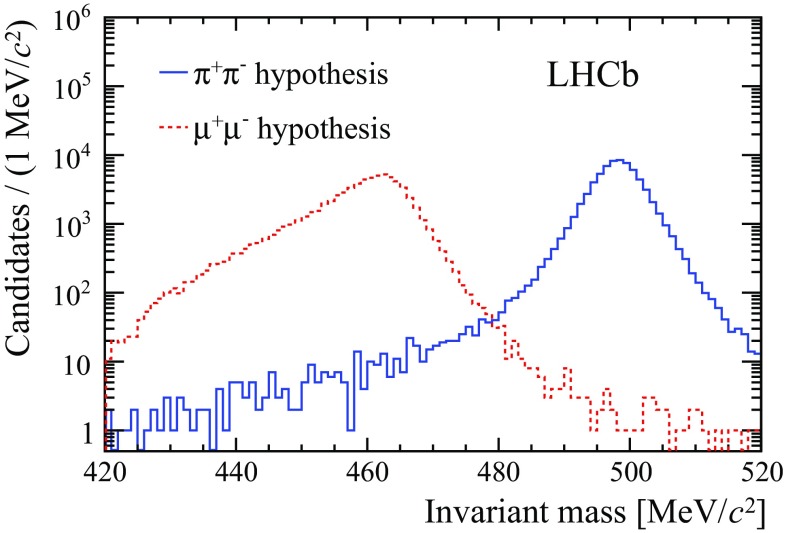
Reconstructed mass for ${{K} ^0_{\mathrm { \scriptscriptstyle S}}} \rightarrow \pi ^+\pi ^-$ decays in trigger-unbiased events, computed assuming the muon (dashed red line) or pion (solid blue line) mass for the final-state tracks. Candidates satisfy the selection criteria described in the text

The preselection reduces the combinatorial background, arising from candidates formed from secondary hadronic collisions in the detector material or from spurious reconstructed SV. The purity of the ${{K} ^0_{\mathrm { \scriptscriptstyle S}}} \rightarrow \pi ^+\pi ^-$ sample used for normalisation, whose mass distribution is shown in Fig. 1, is estimated from a fit to the mass spectrum to be $99.8\%$. The fraction of events with more than one candidate is less than $0.1\%$ for signal and $4\%$ for the normalisation channel, and all candidates are retained. Additional discrimination against backgrounds for the signal mode is achieved through the use of two multivariate discriminants. The first is designed to further suppress combinatorial background, and the second to reduce the number of ${{K} ^0_{\mathrm { \scriptscriptstyle S}}} \rightarrow \pi ^+\pi ^-$ decays in which both pions are misidentified as muons.

After requirements on the output of these discriminants have been applied, the number of signal candidates is obtained by fitting the ${{K} ^0_{\mathrm { \scriptscriptstyle S}}} \rightarrow \mu ^+\mu ^-$ mass spectrum. The number of candidates is converted into a branching fraction using the yield of the ${{K} ^0_{\mathrm { \scriptscriptstyle S}}} \rightarrow \pi ^+\pi ^-$ normalisation mode, and the estimated relative efficiency. Events in the ${K} ^0_{\mathrm { \scriptscriptstyle S}}$ mass region are scrutinised only after fixing the analysis strategy.

## Backgrounds

The ${{K} ^0_{\mathrm { \scriptscriptstyle S}}} \rightarrow \mu ^+\mu ^-$ sample contains two main sources of background. Combinatorial background candidates are expected to exhibit a smooth mass distribution, and can therefore be estimated from the sidebands. The other relevant source of background is due to ${{K} ^0_{\mathrm { \scriptscriptstyle S}}} \rightarrow \pi ^+\pi ^-$ decays where both pions pass the loose muon identification requirements after the trigger stage. This can be due either to ${{\pi } ^+} \!\rightarrow {\mu ^+} {{\nu } _\mu }$ decays or to random association of muon detector hits with the pion trajectory. In such cases the ${K} ^0_{\mathrm { \scriptscriptstyle S}}$ mass, reconstructed with a wrong mass hypothesis for the final-state particles, is underestimated by $39\,{\mathrm {\,MeV\!/}c^2}$ on average, as shown in Fig. 1. Despite the excellent mass resolution, the right-hand tail of the reconstructed mass distribution under the dimuon hypothesis extends into the ${K} ^0_{\mathrm { \scriptscriptstyle S}}$ signal mass range and, given the large branching fraction of the ${{K} ^0_{\mathrm { \scriptscriptstyle S}}} \rightarrow \pi ^+\pi ^-$ mode, constitutes a nonnegligible background. Two multivariate discriminants, based on a boosted decision tree (BDT) algorithm [21, 22], are applied on the preselected candidates to improve the signal discrimination with respect to these backgrounds.

The first discriminant, named hereafter $\text {BDT}_\mathrm{cb}$, aims to reduce the combinatorial background, exploiting the different decay topologies, kinematic spectra and reconstruction qualities of signal and combinatorial candidates. It is optimised separately for each trigger category. The algorithm used for both categories is XGBoost [23], with a learning rate of 0.02 and a maximum depth of 4. The optimal number of estimators is 2000 and 800 for the TOS$_{\mu }$ and TOS$_{\mu \mu }$ trigger categories, respectively. A set of ten input variables is used in $\text {BDT}_\mathrm{cb}$: the ${K} ^0_{\mathrm { \scriptscriptstyle S}}$ $p_{\mathrm { T}}$ and IP, the minimum IP of the two charged tracks, the angle between the positively charged final-state particle in the ${K} ^0_{\mathrm { \scriptscriptstyle S}}$ rest frame and the axis defined by the ${K} ^0_{\mathrm { \scriptscriptstyle S}}$ boost direction, the $\chi ^2$ of the SV fit, the distance of closest approach between the two tracks, an SV isolation variable, defined as the difference in vertex-fit $\chi ^2$ when the next nearest track is included in the vertex fit, and the SV absolute position coordinates. The SV position is particularly important, since a large fraction of the background is found to originate from interactions in the detector material. This set of variables does not distinguish between ${{K} ^0_{\mathrm { \scriptscriptstyle S}}} \rightarrow \mu ^+\mu ^-$ and ${{K} ^0_{\mathrm { \scriptscriptstyle S}}} \rightarrow \pi ^+\pi ^-$ decays as it does not contain quantities related to muon identification and ignores the ${K} ^0_{\mathrm { \scriptscriptstyle S}}$ candidate invariant mass distribution.

The signal training sample for $\text {BDT}_\mathrm{cb}$ is composed of about 11800 (TOS$_{\mu }$) and 2400 (TOS$_{\mu \mu }$) ${{K} ^0_{\mathrm { \scriptscriptstyle S}}} \rightarrow \mu ^+\mu ^-$ simulated candidates passing the trigger and preselection criteria. A signal training sample consisting of ${{K} ^0_{\mathrm { \scriptscriptstyle S}}} \rightarrow \pi ^+\pi ^-$ decays in data is also used as a cross-check, as explained in Sect. 6. The background training sample is made from ${{K} ^0_{\mathrm { \scriptscriptstyle S}}} \rightarrow \mu ^+\mu ^-$ data candidates surviving the trigger and preselection requirements with reconstructed mass in the range $[520,600]\,{\mathrm {\,MeV\!/}c^2}$, and contains about 15000 and 4000 candidates for the TOS$_{\mu }$ and TOS$_{\mu \mu }$ trigger categories, respectively. Since candidates in the same mass region are also used to estimate the residual background, the training is performed using a *k-fold* cross-validation technique [24] to avoid any possible effect of overtraining.

A loose requirement on the $\text {BDT}_\mathrm{cb}$ output is applied to suppress the combinatorial background. The cut is chosen to remove 99% of the background training candidates. The corresponding signal efficiency is about 56 and $66\%$ for the TOS$_{\mu }$ and TOS$_{\mu \mu }$ trigger categories, respectively. To exploit further the information provided by the discriminant, the candidates surviving this requirement are allocated to ten bins according to their $\text {BDT}_\mathrm{cb}$ value, with bounds defined in order to have approximately equal population of signal training candidates in each bin.

The background from misidentified ${{K} ^0_{\mathrm { \scriptscriptstyle S}}} \rightarrow \pi ^+\pi ^-$ decays is further reduced with the second multivariate discriminant, called $\text {BDT}_\mu$. Its input includes the position, time and number of detector hits around the extrapolated track position to each muon detector station, a global match $\chi ^2$ between the muon hit positions and the track extrapolation, and other variables related to the tracking and the response of the RICH and calorimeter detectors.

To train the $\text {BDT}_\mu$ discriminant, highly pure samples of 1.2 million pions and 0.68 million muons are obtained from TIS-triggered ${{K} ^0_{\mathrm { \scriptscriptstyle S}}} \rightarrow \pi ^+\pi ^-$ and $B^+\rightarrow J/\psi K^+$ decays, respectively. In the latter case, a probe muon from the ${J /\psi }$ is required to be TIS at all trigger stages, while stringent muon identification requirements are set on the other muon, reaching an estimated purity for muons above $99.9\%$. The multivariate AdaBoost algorithm implemented in the TMVA package [25] is used, with 850 trees and a maximum depth of 3. Before using it in the $\text {BDT}_\mu$ training, the muon sample is weighted to have the same two-dimensional distribution in $p$ and $p_{\mathrm { T}}$ as the pion sample, as well as the same distribution of number of tracks in the event. This is to prevent the $\text {BDT}_\mu$ from discriminating pions and muons using these variables, which are included in the input because of their strong correlation with the identification variables. Weighting also allows optimisation of the discrimination power for the kinematic spectrum relevant to this search.

The level of misidentification of the discriminant for a pion from ${{K} ^0_{\mathrm { \scriptscriptstyle S}}} \rightarrow \pi ^+\pi ^-$ decay is found to be 0.4% for $90\%$ muon efficiency. This reduces the level of double misidentification background, for a given efficiency, by about a factor of four with respect to the discriminant used in the previous publication [6], which was not tuned specifically for ${{K} ^0_{\mathrm { \scriptscriptstyle S}}} \rightarrow \mu ^+\mu ^-$ searches.

The $\text {BDT}_\mu$ discriminant is trained using half of the $B^+\rightarrow J/\psi K^+$ sample, while the other half is used to evaluate the muon identification efficiency as a function of ($p$, $p_{\mathrm { T}}$). These values are used to compute the efficiency of a $\text {BDT}_\mu$ requirement on the candidate ${{K} ^0_{\mathrm { \scriptscriptstyle S}}} \rightarrow \mu ^+\mu ^-$ decays after selection and trigger requirements, in each bin of the $\text {BDT}_\mathrm{cb}$ discriminant. The muon spectra assumed in this calculation are obtained from simulated decays, weighted to better reproduce the ${K} ^0_{\mathrm { \scriptscriptstyle S}}$ $p_{\mathrm { T}}$ spectrum observed in ${{K} ^0_{\mathrm { \scriptscriptstyle S}}} \rightarrow \pi ^+\pi ^-$ candidates.

The $\text {BDT}_\mu$ requirement on the signal candidates is optimised by maximising the figure of merit [26] $\epsilon _{\mu \mathrm{{ID}}}/(\sqrt{N_\mathrm{bg}}+a/2)$, with $a=3$, where $\epsilon _{\mu \mathrm{{ID}}}$ is the signal efficiency and $N_\mathrm{bg}$ the expected background yield. The latter is estimated from a fit to the mass distribution, after removing candidates in the range $[492,504]\,{\mathrm {\,MeV\!/}c^2}$ around the ${K} ^0_{\mathrm { \scriptscriptstyle S}}$ mass, and extrapolating the result into this region. In the fit, the contribution of ${{K} ^0_{\mathrm { \scriptscriptstyle S}}} \rightarrow \pi ^+\pi ^-$ decays is modelled with a Crystal Ball function [27] and the combinatorial background with an exponential function, where all the parameters are left free to vary. This optimisation is performed independently for the two trigger categories, with no significant difference found as a function of the $\text {BDT}_\mathrm{cb}$ bin. The optimal threshold corresponds to a signal efficiency of $\epsilon _{\mu \mathrm{{ID}}} \sim 98\%$ in both cases.

Other possible sources of background have been explored and found to give negligible contribution to this search. The irreducible background due to ${{K} ^0_{\mathrm { \scriptscriptstyle L}}} \!\rightarrow {\mu ^+\mu ^-}$ decays and from ${K} ^0_{\mathrm { \scriptscriptstyle S}}$–${K} ^0_{\mathrm { \scriptscriptstyle L}}$ interference is evaluated from the known ${{K} ^0_{\mathrm { \scriptscriptstyle L}}} \rightarrow \mu ^+\mu ^-$ branching fraction and lifetime, and by studying the decay-time dependence of the selection efficiency for ${{K} ^0_{\mathrm { \scriptscriptstyle S}}} \rightarrow \pi ^+\pi ^-$ decays in data. The yield from this background becomes comparable to the signal for a branching fraction lower than $2 \times 10^{-11}$, which is well below the sensitivity of this search.

Semileptonic ${{\overline{K}{}} {}^0} \!\rightarrow {{\pi } ^+} {\mu ^-} {{\overline{\nu }} _\mu }$ decays with pion misidentification provide another possible source of background. Simulated events, where the pion is forced to decay to $\mu \nu$ within the detector, are used to determine the efficiency of the offline selection requirements. No event survives the trigger selection. Under the very conservative hypothesis that the trigger efficiency is the same as in ${{K} ^0_{\mathrm { \scriptscriptstyle S}}} \rightarrow \mu ^+\mu ^-$ decays, the expected yields from both ${K} ^0_{\mathrm { \scriptscriptstyle L}}$ and ${K} ^0_{\mathrm { \scriptscriptstyle S}}$ semileptonic decays are negligible.

Decays including a dimuon from resonances, like $\omega \!\rightarrow {{\pi } ^0} {\mu ^+\mu ^-}$ and $\eta \!\rightarrow {\mu ^+\mu ^-} \gamma$, do not produce peaking structures in the mass distribution, and are accounted for in the combinatorial background.

**Table 1:** Values of the single candidate sensitivity $\alpha _{ij}$ and the number of candidates $N^{K}_{ij}$ compatible with the ${K} ^0_{\mathrm { \scriptscriptstyle S}}$ mass (reconstructed mass in the range $[492,504]\,{\mathrm {\,MeV\!/}c^2}$), for each $\text {BDT}_\mathrm{cb}$ bin *i* and trigger category *j*. Only statistical uncertainties are given. The first uncertainty is uncorrelated, while the second is fully correlated among the $\text {BDT}_\mathrm{cb}$ bins of the same trigger category

Bin *i*	$\alpha _{i \text {TOS}_\mu } (\times 10^{-10})$	$\alpha _{i \text {TOS}_{\mu \mu }} (\times 10^{-9})$	$N^{K}_{i \text {TOS}_\mu }$	$N^{K}_{i \text {TOS}_{\mu \mu }}$
1	$7.48 \pm 0.84 \pm 0.16$	$5.30 \pm 0.72 \pm 0.12$	49	13
2	$7.72 \pm 0.87 \pm 0.17$	$4.71 \pm 0.63 \pm 0.10$	28	9
3	$7.85 \pm 0.89 \pm 0.18$	$4.88 \pm 0.65 \pm 0.11$	9	14
4	$7.93 \pm 0.89 \pm 0.19$	$4.66 \pm 0.62 \pm 0.10$	18	10
5	$7.53 \pm 0.85 \pm 0.18$	$4.65 \pm 0.61 \pm 0.10$	6	3
6	$7.78 \pm 0.88 \pm 0.19$	$4.95 \pm 0.66 \pm 0.11$	2	2
7	$7.56 \pm 0.85 \pm 0.19$	$4.60 \pm 0.61 \pm 0.10$	3	1
8	$7.90 \pm 0.89 \pm 0.19$	$5.00 \pm 0.67 \pm 0.11$	2	1
9	$7.81 \pm 0.88 \pm 0.18$	$4.72 \pm 0.63 \pm 0.11$	1	1
10	$7.75 \pm 0.87 \pm 0.17$	$4.66 \pm 0.62 \pm 0.11$	0	0

## Search sensitivity

The observed number of ${{K} ^0_{\mathrm { \scriptscriptstyle S}}} \rightarrow \mu ^+\mu ^-$ candidates is converted into a branching fraction using the normalisation mode and its precisely known branching fraction $\mathcal{B}({{K} ^0_{\mathrm { \scriptscriptstyle S}}} \rightarrow \pi ^+\pi ^-) = 0.6920\pm 0.0005$ [4]. The computation is made in every $\text {BDT}_\mathrm{cb}$ bin *i* and trigger category *j* as follows

1 $$\begin{aligned}&\mathcal{B}({{K} ^0_{\mathrm { \scriptscriptstyle S}}} \rightarrow \mu ^+\mu ^-) =\mathcal{B}({{K} ^0_{\mathrm { \scriptscriptstyle S}}} \rightarrow \pi ^+\pi ^-) \cdot \frac{ \epsilon ^{\pi \pi }}{\epsilon ^{\mu \mu }_{ij}} \cdot \frac{N^{\mu \mu }_{ij}}{N^{\pi \pi }}\nonumber \\&\quad \equiv \alpha _{ij} N^{\mu \mu }_{ij}, \end{aligned}$$

where $N^{\mu \mu }_{ij}$ and $N^{\pi \pi }$ denote the background-subtracted yields for the signal and normalisation modes, respectively. The total selection efficiencies $\epsilon$ can be factorised as

2 $$\begin{aligned} \frac{\epsilon ^{\pi \pi }}{\epsilon ^{\mu \mu }_{ij}} = \frac{\epsilon ^{\pi \pi }_\mathrm{{sel}}}{\epsilon ^{\mu \mu }_\mathrm{{sel}}} \times \frac{\epsilon ^{\pi \pi }_{\mathrm{{trig}}}}{\epsilon ^{\mu \mu }_{\mathrm{{trig}};j}} \times \frac{1}{\epsilon ^{\mu \mu }_{{\text {BDT}};ij}} \times \frac{1}{\epsilon _{\mu \mathrm{{ID}};ij}}. \end{aligned}$$

The first factor refers to the offline selection requirements, which are applied identically to both modes and cancel to first order in the ratio; the residual difference is mainly due to the different interaction cross-sections for pions and muons with the detector material, and is estimated from simulation. The second factor is the ratio of trigger efficiencies; the efficiency for the signal is determined from simulation, with its systematic uncertainty estimated from data-driven checks, while that for the normalisation mode is the prescale factor of the random trigger used to select ${{K} ^0_{\mathrm { \scriptscriptstyle S}}} \rightarrow \pi ^+\pi ^-$, $(9.38 \pm 1.01)\times 10^{-8}$. The third factor reflects the fraction of candidates in each $\text {BDT}_\mathrm{cb}$ bin, and is also determined from simulation. Finally, the efficiency of the $\text {BDT}_\mu$ requirement is obtained from the $B^+\rightarrow J/\psi K^+$ calibration sample described in Sect. 4, for each $\text {BDT}_\mathrm{cb}$ bin and trigger category.

To account for the difference between the kaon $p_{\mathrm { T}}$ spectra observed in the ${{K} ^0_{\mathrm { \scriptscriptstyle S}}} \rightarrow \pi ^+\pi ^-$ decays in data and simulation, all efficiencies obtained from simulation are computed in six roughly equally populated $p_{\mathrm { T}}$ bins. A weighted average of the efficiencies is then performed, where the weights are determined from the yields in each bin observed in data for ${{K} ^0_{\mathrm { \scriptscriptstyle S}}} \rightarrow \pi ^+\pi ^-$ candidates.

The resulting values for the single candidate sensitivity $\alpha _{ij}$ are reported in Table 1. The quoted uncertainties are statistical only. They are separated between the uncertainty on $\epsilon ^{\mu \mu }_{{\text {BDT}};ij}$, due to the limited statistics of simulated data and uncorrelated among $\text {BDT}_\mathrm{cb}$ bins, and all the other statistical uncertainties, which are conservatively considered as fully correlated among bins within the same trigger category. Table 1 also presents the number of candidates around the ${K} ^0_{\mathrm { \scriptscriptstyle S}}$ mass. The separation between signal and background is presented in Sect. 7.

## Systematic uncertainties

Several systematic effects, summarised in Table 2, contribute to the uncertainty on the normalisation factors. Tracking efficiencies are not perfectly reproduced in simulated events. Corrections based on a $${{J /\psi }} \!\rightarrow {\mu ^+\mu ^-} $$ data control sample are determined as a function of the muon $$p $$ and $$\eta $$. The average effect of these corrections on the ratio $$\epsilon ^{\pi \pi }_\mathrm{sel}/\epsilon ^{\mu \mu }_\mathrm{sel}$$ and its standard deviation, added in quadrature, leads to a systematic uncertainty of $$0.4\%$$.

**Table 2:** Relevant systematic uncertainties on the branching fraction. They are separated, using horizontal lines, into relative uncertainties on (i) $\alpha _{ij}$, (ii) on the signal yield from the signal model used in the mass fit, and (iii) on the branching fraction, obtained combining the two categories, from the background model

Source	TOS$_{\mu }$	TOS$_{\mu \mu }$
Tracking (%)	0.4	0.4
Selection (%)	1.9	1.8
Trigger (%)	8.1	11.5
${K} ^0_{\mathrm { \scriptscriptstyle S}}$ $p_{\mathrm { T}}$ spectrum (%)	4.3	4.3
Muon identification (%)	0.2	0.3
Signal mass shape (%)	0.8	0.8
Background shape (%)	0.9	

The distributions of all variables relevant to the selection are compared in data and simulation for ${{K} ^0_{\mathrm { \scriptscriptstyle S}}} \rightarrow \pi ^+\pi ^-$ decays. The largest differences are found in the kaon $p_{\mathrm { T}}$ and its decay vertex radial position. The effect on $\epsilon ^{\pi \pi }_\mathrm{sel}/\epsilon ^{\mu \mu }_\mathrm{sel}$ of applying a two-dimensional weight to account for these discrepancies is taken as a systematic uncertainty, and amounts to a relative 1.9 and $1.8\%$ for the TOS$_{\mu }$ and TOS$_{\mu \mu }$ trigger categories, respectively.

The difference between data and simulation in the kaon $p_{\mathrm { T}}$ spectrum could also affect the other factors in the computation of $\alpha _{ij}$. An additional uncertainty is assigned by repeating the whole calculation with a finer binning in $p_{\mathrm { T}}$. Due to the limited size of the data samples, this is possible only in the TOS$_{\mu }$ category. The average relative change in $\alpha _{ij}$, $4.3\%$, is assigned as an uncertainty for both categories.

A specific cross-check is performed to validate the efficiencies predicted by the simulation for the $\text {BDT}_\mathrm{cb}$ requirements. An alternative discriminant is made using a signal training sample consisting of trigger-unbiased ${{K} ^0_{\mathrm { \scriptscriptstyle S}}} \rightarrow \pi ^+\pi ^-$ decays, selected with additional kinematic criteria which mimic the effect of the muon trigger selections. The distributions of this alternative discriminant in data and simulation are found to agree within the statistical uncertainty, and no systematic uncertainty is assigned.

The uncertainty due to the simulation of TOS selections in the first two trigger stages is assessed by comparing the trigger efficiency in simulation and data, using a control sample of ${{B} ^+} \!\rightarrow {{J /\psi }} {{K} ^+}$ decays. The resulting relative differences, $8.1\%$ for TOS$_{\mu }$ and $11.5\%$ for TOS$_{\mu \mu }$, are assigned as systematic uncertainties. No uncertainty is considered for the selection in the last trigger stage, which is based on the same offline kinematic variables used in the selection, for which a systematic uncertainty is already assigned.

The uncertainty on $\epsilon _{\mu \mathrm{{ID}};ij}$ is estimated from half the difference between the values obtained with and without the weighting of the ${{B} ^+} \!\rightarrow {{J /\psi }} {{K} ^+}$ sample used in the determination of the muon identification efficiency. This results in an uncertainty of 0.2 and 0.3% for the TOS$_{\mu }$ and TOS$_{\mu \mu }$ categories, respectively, which is comparable to the statistical uncertainties on these efficiencies due to the limited size of the ${{B} ^+} \!\rightarrow {{J /\psi }} {{K} ^+}$ samples.

Systematic uncertainties on the signal yields $N^{\mu \mu }_{ij}$ are related to the assumed models for the reconstructed ${K} ^0_{\mathrm { \scriptscriptstyle S}}$ mass distribution, determined from simulation. Possible discrepancies from the shape in data are estimated by comparing the shape of the invariant mass distribution in data and simulation for ${{K} ^0_{\mathrm { \scriptscriptstyle S}}} \rightarrow \pi ^+\pi ^-$ decays, leading to a relative $0.8\%$ systematic uncertainty on the signal yield. The final fit for the determination of the branching fraction is performed with two different background models, as discussed in Sect. 7. This leads to a relative variation on the branching fraction of 0.9%, which is assigned as a systematic uncertainty.

**Fig. 2:**
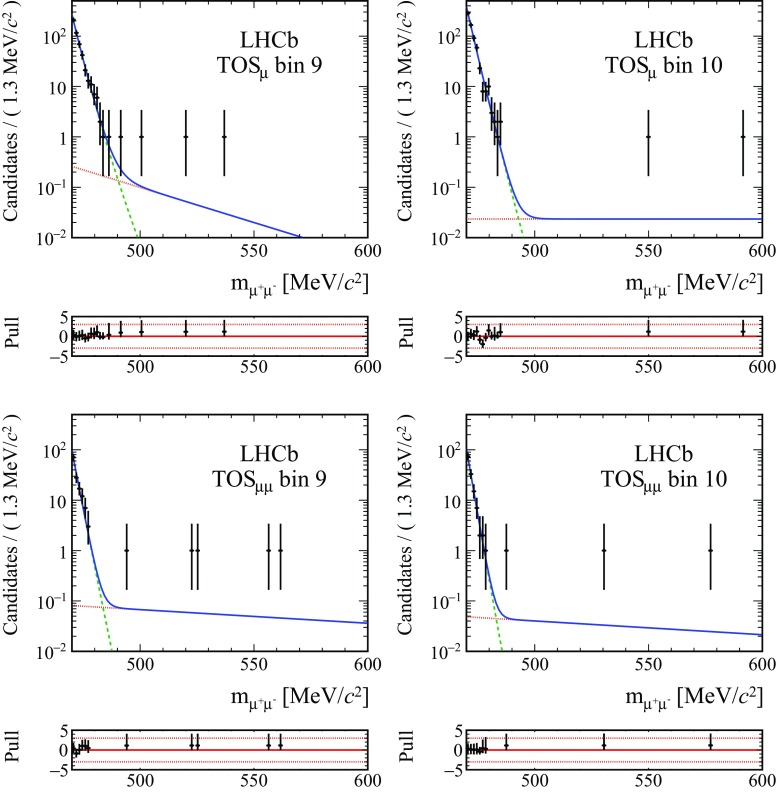
Fits to the reconstructed kaon mass distributions, for the two most sensitive $\text {BDT}_\mathrm{cb}$ bins in the two trigger categories, TOS$_{\mu }$ and TOS$_{\mu \mu }$. The fitted model is shown as the solid blue line, while the combinatorial background and ${{K} ^0_{\mathrm { \scriptscriptstyle S}}} \rightarrow \pi ^+\pi ^-$ double misidentification are overlaid with dotted red and dashed green lines, respectively. For each fit, the pulls are shown on the lower smaller plots

## Results

The $\mu ^+\mu ^-$ mass distribution of the signal candidates is fitted in the range $[470,600]\,{\mathrm {\,MeV\!/}c^2}$ to determine the signal and background yield in each trigger category and $\text {BDT}_\mathrm{cb}$ bin. The mass distribution of simulated signal candidates is best described by a Hypatia function [28]. Its parameters are determined from simulation and fixed in the fit to data. In the background model, a power law function describes the tail of the double-misidentification background from ${{K} ^0_{\mathrm { \scriptscriptstyle S}}} \rightarrow \pi ^+\pi ^-$ decays, affecting the mass region below the ${K} ^0_{\mathrm { \scriptscriptstyle S}}$ mass, while the combinatorial background mass distribution is described by an exponential function. The background model is validated on simulation, and its parameters are left free in the fit to data to account for possible discrepancies. An alternative combinatorial background shape, based on a linear function, is used instead of the exponential function to determine a systematic uncertainty due to the choice of the background shape. The signal yields in each BDT bin for the two trigger categories are all compatible with the absence of ${{K} ^0_{\mathrm { \scriptscriptstyle S}}} \rightarrow \mu ^+\mu ^-$ candidates. The $\mu ^+\mu ^-$ invariant mass distributions for the two highest $\text {BDT}_\mathrm{cb}$ bins, which exhibit the best signal-to-background ratio and therefore the best sensitivity for a discovery, are shown in Fig. 2.

**Fig. 3:**
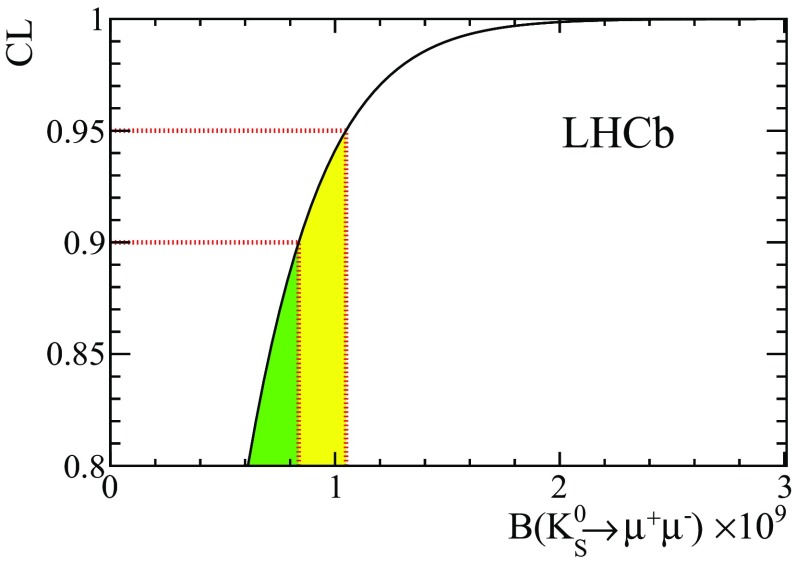
Confidence level of exclusion for each value of the ${{K} ^0_{\mathrm { \scriptscriptstyle S}}} \rightarrow \mu ^+\mu ^-$ branching fraction. The regions corresponding to $90\%$ and $95\%$ CL are emphasised in green (dark shading) and yellow (light shading), respectively

A simultaneous maximum likelihood fit to the dimuon mass in all $\text {BDT}_\mathrm{cb}$ bins is performed, using the values of $\alpha _{ij}$ given in Table 1 and the normalization channel yield $N^{\pi \pi }$, to determine the branching fraction. The ${{K} ^0_{\mathrm { \scriptscriptstyle S}}} \rightarrow \pi ^+\pi ^-$ candidates are counted within the mass region $[460,\,530]\,$ ${\mathrm {\,MeV\!/}c^2}$, leading to $N^{\pi \pi }=70\,318\pm 265$. The quoted systematic uncertainties are included in the likelihood computation as nuisance parameters with Gaussian uncertainties. A posterior probability is obtained by multiplying the likelihood by a prior density, which is computed as the product of the likelihood from the 2011 analysis and a flat prior over the positive range of the branching fraction. Limits are obtained by integrating $90\%~(95\%)$ of the area of the posterior probability distribution provided by the fit, as shown in Fig. 3. Due to the much larger sensitivity achieved with the 2012 data, the inclusion of the 2011 data result does not have a significant effect on the final limit, and a uniform prior would have provided very similar results. The expected upper limit, and the compatibility with background-only hypothesis have been computed by means of pseudoexperiments, where samples of background events are randomly generated according to the mass distribution obtained by the best fit to data. The median expected upper limit and its $\pm 1\sigma$ range is $\mathcal{B}({{K} ^0_{\mathrm { \scriptscriptstyle S}}} \rightarrow \mu ^+\mu ^-) < 0.95^{+0.42}_{-0.27} ~(1.17^{+0.45}_{-0.31}) \times 10^{-9}~\text {at}~90\%~(95\%)~\text {CL}$. The observed limit is

$$\begin{aligned} \mathcal{B}({{K} ^0_{\mathrm { \scriptscriptstyle S}}} \rightarrow \mu ^+\mu ^-) < 0.8~(1.0) \times 10^{-9}~\text {at}~90\%~(95\%)~\text {CL}. \end{aligned}$$

The compatibility of the experimental measurement with the background-only model, expressed in terms of p value is 0.52.

In conclusion, a search for the ${{K} ^0_{\mathrm { \scriptscriptstyle S}}} \rightarrow \mu ^+\mu ^-$ decay based on a data sample corresponding to an integrated luminosity of $3\,\text{ fb }^{-1}$ of proton-proton collisions, collected by the LHCb experiment at centre-of-mass energies $\sqrt{s}=7$ and 8$\mathrm {\,TeV}$, improves the upper limit for this decay by a factor 11 with respect to the previous search published by LHCb  [6], which is superseded by this result.

## Footnotes

1 The inclusion of charge-conjugate processes is implied throughout.
